# Brood size, food availability, and body size affects male care decisions and offspring performance

**DOI:** 10.1002/ece3.10183

**Published:** 2023-06-08

**Authors:** Jacqueline Sahm, Taina Conrad, Larissa Scheu, Sandra Steiger

**Affiliations:** ^1^ Department of Evolutionary Animal Ecology University of Bayreuth Bayreuth Germany

**Keywords:** burying beetles, flexible parenting, male care, *Nicrophorus*, parental investment

## Abstract

Parental care strategies do not only vary greatly across species, but also within species there can be substantial between‐ and within‐individual variation in parental care behavior. To better understand the evolution of care strategies, it is crucial to determine how and when parents modify their behavior in response to internal as well as environmental factors. Here, we investigated the effect of brood size, resource size and an individual's quality on care strategies of uniparental males and examined the downstream consequences on offspring performance in the burying beetle *Nicrophorus vespilloides*. Burying beetles breed on small vertebrate cadavers and, on average, males invest much less in care than females. Nevertheless, we found that uniparentally caring males were responsive to their social and non‐social environment and adjusted the amount as well as the type of care to the size of the brood, the size of the cadaver and their own body size. Additionally, we show that the care strategies affected offspring performance. Specifically, males that cared longer had larger and more surviving larvae. Our results add to our understanding of plastic parenting strategies by showing that even the sex that provides less care can evolve a very flexible care behavior.

## Graphical abstract

Parental care strategies can differ between and within individuals. To better understand the evolution of flexible care strategies, we investigated the effect of internal as well as environmental factors on care strategies of uniparental males and examined the downstream consequences on offspring performance in the burying beetle Nicrophorus vespilloides. We found that uniparentally caring males were very responsive to their social and non‐social environment showing that even the sex that provides less care can evolve a very flexible care behavior.

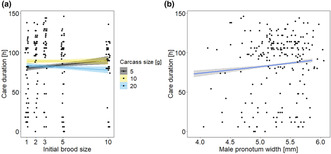

## 1 INTRODUCTION

Parental care, which is a taxonomically widespread strategy, comprises all parental traits that increase the fitness of a parent's offspring (Meunier et al., 2022; Smiseth et al., 2012; Trivers, 1972; Wong et al., 2013). Parental care can occur before or after the birth of offspring and includes various behaviors, such as the protection of offspring from predators, the maintenance of a favorable microenvironment and offspring provisioning (Balshine, 2012; Smiseth et al., 2012; Trumbo, 2012) amongst others. Across the animal kingdom, female care is much more widespread than male care (Clutton‐Brock, 1991) and even in biparental families, males frequently invest less than females and desert the brood earlier or with a higher probability. The reason for this asymmetry is thought to lie in sex differences in the strength of sexual selection, in the association with the embryo, and in the certainty of parentage (Liker et al., 2015; Møller, 1988; Møller & Birkhead, 1993; Queller, 1997; Royle et al., 2016; Westneat & Sargent, 1996; Westneat & Sherman, 1993). In some species, for example, mothers have a relatively high certainty about their maternity, whereas males – especially in species with internal fertilization and multiple mating females – are often uncertain about their paternity (Trivers, 1972; Westneat & Sherman, 1993). Since parental care usually comes with costs to parents and ultimately lowers future reproductive success, offspring desertion can be considered as an investment in re‐mating and future young (Székely et al., 1996). The decision how much to invest in a current brood has been predicted and/or empirically shown to depend on a multitude of factors, such as food availability, predation risk, paternity certainty, the value of the brood (e.g., brood size), parental quality (e.g., body size) or the partner's investment decisions (Erikstad et al., 1997; Hõrak et al., 1999; Magrath et al., 2007; Meunier & Kölliker, 2012; Neff, 2003; Parejo & Danchin, 2006; Pilakouta et al., 2015; Royle et al., 2014; Wright & Cuthill, 1990b). A range of studies have shown, for example, that parents compensate – at least partially – for the loss of their partner or adjust care behavior according to brood size (Griggio & Pilastro, 2007; Harrison et al., 2009; Wang, Ma, et al., 2021; Wright & Cuthill, 1990a). Also, attributes of the parent itself, such as body size or condition, have been shown to impact care decisions. For example, in snow petrels (*Pagodroma nivea*), parents in good body conditions guard their chicks longer (Tveraa & Christensen, 2002) and in pine engraver beetles (*Ips pini*) larger males leave their brood earlier than smaller ones (Robertson & Roitberg, 1998). From an ultimate perspective, parents are expected to modify their care behavior according to the costs and benefits of care, providing more care when the benefit is higher and providing less care when the costs are higher (Alonso‐Alvarez & Velando, 2012). However, in families in which males usually provide less care than females, males might not be selected to be as responsive as females to the social or non‐social environment they experience. Males might also differ in their care behavior due to other factors. For example, because males do not have the ability to increase brood size by laying additional eggs, males might abandon small broods more often than females. Although a range of studies have examined female care behavior after mate loss (Cantarero et al., 2019; Fetherston et al., 1994; Markman et al., 1995; Sakaluk et al., 1998; Sanz et al., 2000; Smiseth et al., 2005; Wang, Ma, et al., 2021), only few studies have considered male investment decisions. Thus, it remains unclear how responsive they are towards environmental and internal cues and how they react when confronted with small broods in the absence of their female partner.

We addressed this knowledge gap using the burying beetle *Nicrophorus vespilloides* as a model organism. Burying beetles are an ideal system to examine paternal investment decisions as they provide elaborate biparental care (Eggert, 1992; Eggert & Müller, 1997; Trumbo, 1991; Wilson & Fudge, 1984), uniparental female care (Scott & Traniello, 1990; Smiseth et al., 2005; Steiger, 2013), as well as uniparental male care (Luzar et al., 2017; Parker et al., 2015; Scott, 1989; Trumbo & Fernandez, 1995; Ward et al., 2009). *Nicrophorus* beetles use small vertebrate cadavers as a breeding resource (Eggert & Müller, 1997; Pukowski, 1933; Royle et al., 2013; Scott, 1998b). Carrion is a nutrient rich but scarce resource leading to a high competition between con‐ and allospecific beetles for the monopolization of the resource. In the competition over carrion, the body size of beetles is a good predictor of the conflict outcome with larger individuals usually winning the contests (Otronen, 1988; Robertson, 1993; Trumbo, 1990; Trumbo, 1994). During breeding, parents transform the cadaver into an edible nursery for their offspring (Duarte et al., 2021; Pukowski, 1933; Royle et al., 2013; Scott, 1998b; Trumbo & Robinson, 2004). Parents remove fur or feathers, treat the carcass with antimicrobial secretions, and create a feeding cavity, within which larvae aggregate to either feed themselves or to get fed by the parents (Müller et al., 1998; Shukla et al., 2018; Smiseth, Bu, et al., 2003; Smiseth, Darwell, & Moore, 2003). When caring biparentally, females predominantly provide direct care (Scott & Traniello, 1990; Smiseth et al., 2005; Smiseth & Moore, 2004b; Walling et al., 2008), whereas males often focus on indirect care i.e., carcass maintenance and defense (Fetherston et al., 1990; Trumbo, 1991, 2007). Additionally, it is known that males usually desert earlier than females (Bartlett, 1988; Fetherston et al., 1990; Müller et al., 2007; Parker et al., 2015; Ratz et al., 2021; Royle et al., 2014; Scott, 1998a; Ward et al., 2009). However, if the female deserts or dies, males are able to compensate for the loss of their partner (Bartlett, 1988; Jenkins et al., 2000; Müller et al., 1998; Scott, 1989; Smiseth et al., 2005; Trumbo & Fernandez, 1995). Even though males can adjust their care behavior based on the presence and absence of a partner, it is still unclear whether males are able to perceive and respond to other environmental factors, such as brood or resource size, or whether they base their care decisions on their own quality. From females we know that they are quite responsive to their breeding environment (Royle & Hopwood, 2017). For example, if females face small brood sizes – due to hatching failure or predation ‐ they frequently respond by producing a second egg clutch (Müller, 1987; Sahm et al., 2022). They also take into account carcass size and lay additional eggs more frequently when monopolizing larger carcasses (Sahm et al., 2022). Although males are unable to increase the initial brood size, they still might show plastic behavioral responses towards broods of different sizes. Since *N. vespilloides* larvae can self‐feed from the carrion resource and can partially survive in the absence of parents (Capodeanu‐Nägler et al., 2016), males might, for example, abandon small broods or spend less time caring for them. Especially on large carcasses, it is also possible that they kill small broods to preserve the carcass and attract a new female via their sex pheromone (Eggert & Müller, 1989; Chemnitz et al., 2017). In fact, due to a high paternity uncertainty – as females mate multiple times in burying beetles (House et al., 2008; Müller et al., 2007; Müller & Eggert, 1989) ‐ males might be more likely to desert a given breeding attempt rather than care for few offspring. However, up until now empirical studies are missing about the effect of brood and resource size on the care decisions of male burying beetles. Furthermore, it is unclear whether males base their decisions on their own body size. Male body size likely affects the cost–benefit ratio of care and should therefore have an impact on paternal investment decisions.

Our study aimed to tackle the question of whether uniparental male care strategies are influenced by initial brood and carcass size as well as their own quality (i.e., body size) in the burying beetle *Nicrophorus vespilloides*. Additionally, we tested whether the care decisions of males affect offspring performance. Similar to a previous study (Sahm et al., 2022) that focused on female care strategies (under uni‐ and biparental conditions), we adopted a 5 × 3 factorial design and provided males with 1, 2, 3, 5, or 10 larvae and 5, 10 or 20 g carcasses. We used small brood sizes, because we were especially interested in the response of males when confronted with one or few offspring. Since body size shows high variation between individuals, we used the natural variation in male size instead of manipulating their size in our experiment (Steiger, 2013). As response variables, we examined whether males cared for or deserted the brood and the time invested in caring. Furthermore, we evaluated offspring performance by recording the growth and survival of larvae. We predicted that single males would be more likely to stay and invest more time in larger broods and on larger carcasses. Since males of larger size have a higher chance of defending a carcass, and – similar to females (Steiger, 2013) – might be able to raise larger larvae or suffer less costs from caring, we predicted that they would desert the brood less frequently or care longer than smaller males. Additionally, we predicted that a male's care strategy should affect offspring performance, with males that invest more time in brood care raising more or heavier offspring.

## 2 MATERIAL AND METHODS

### 2.1 Origin and husbandry of burying beetles

This study was conducted using an outbred laboratory population of *Nicrophorus vespilloides* kept at the University of Bayreuth, Germany. Experimental beetles belonged to the 5th generation of *N. vespilloides* descending from wild caught beetles captured in a forest near Bayreuth, Germany, in summer 2018. Beetles were held in small plastic containers (10 × 10 × 6 cm) filled with moist peat. Containers were stored in a climate chamber with a 16:8 dark: light cycle at 20°C and fed twice a week using sliced mealworms (*Tenebrio molitor*).

### 2.2 Experimental design and procedures

We investigated the effect of initial brood size and carcass size on the behavior of *N. vespilloides* males using a 5 × 3 factorial design: We manipulated the initial brood size (1, 2, 3, 5 or 10 larvae) as well as the size of a given mouse cadaver (~5, 10 or 20 g). We set up 16 pairs of beetles per treatment group using beetles aged between 20‐ and 30‐day leading to a final sample size of 240 pairs. Since 27 replicates failed to produce any eggs, we conducted our analysis with a final sample size of *N* = 213.

At first, we paired unrelated virgin males and females in plastic containers (9.5 × 9.5 × 5.5 cm) each filled one‐third with moist peat. To ensure sperm supply and egg fertilization, we allowed male beetles to mate multiple times with their female over a 72‐h period. Then we assigned a prior weighted mouse cadaver to each pair (mean ± SD, 5 g: 5.79 g ± 0.96; 10 g: 10.1 g ± 1.27; 20 g: 20.52 g ± 1.38). Since the aim of our study was to analyze the investment behavior of uniparental males, we removed the female partner after a defined period of egg laying, i.e., 48 h after the pairs were provisioned with mice cadavers. To manipulate the initial brood size and to ensure that larvae can hatch in isolation, we separated the male beetles from their eggs, placing them, along with their respective carcass, in a new, equal plastic box filled with moist peat. Over a 48‐h period we checked the old boxes for newly hatched larvae at least every 4 h day and night. We pooled synchronously hatching larvae in petri dishes containing a wet paper towel before randomly assigning them to the different treatment groups: Males received either 1, 2, 3, 5 or 10 larvae as initial brood size. Since *N. vespilloides* is unable to differentiate between their own and unrelated foster offspring based on direct recognition cues (Müller & Eggert, 1990), we were able to provide males with larvae of mixed parentage. Burying beetles frequently kill larvae that arrive sooner on the carcass than their own larvae would (Müller & Eggert, 1990), therefore, we only provided males with an initial brood once their own larvae had hatched. The manipulation of brood size and the use of larvae of mixed parentage is a well‐established protocol in burying beetles (Engel et al., 2016; Oldekop et al., 2007; Rauter & Moore, 1999; Sahm et al., 2022).

To analyze how males respond to the different‐sized broods, we checked the respective containers every 6 h over a 72‐h period following the assignment of larvae to the males and subsequently every 8 h for another 3‐day period. Finally, we observed the treatments every 12 h until the larvae dispersed from the carrion for pupation. During each observation, we recorded whether the male was off or on the carcass and whether he was at or inside the feeding cavity. Similar to Moss and Moore (2021), we categorized all instances, in which the male associated with the larvae and therefore was at or inside the feeding cavity as direct care and all instances in which males occurred at the carcass without contact to the larvae as indirect care. If the male left the carcass for more than 12 consecutive hours before the larvae dispersed from the carcass, we defined his behavior as offspring desertion. As soon as larvae dispersed, we determined the pronotum width of males as a measure of body size. To investigate how male behavior and size affects the survival and fitness of offspring we counted and weighed the dispersing larvae before we placed them in a new box containing moist peat, allowing them to pupate. Lastly, we recorded the number of eclosed adults.

### 2.3 Statistical analysis

All data were analyzed and plotted using R version 3.6.1. We first examined whether initial brood size, carcass size, the interaction between initial brood and carcass size and a male's body size affected the care decisions of *N. vespilloides* males. Initial brood size and body size were entered as continuous variables and carcass size as a category. As response variables, we used (1) the decision of males to either desert or care for a given brood, (2) the duration of care, (3) the absolute amount of direct care (the number of observations in which the male was found at or in the feeding cavity), and (4) the absolute amount of indirect care (the number of observations in which the male was found at the carcass but not at or in the feeding cavity). The variable 1 was fitted to a generalized linear model (GLM) with a binomial distribution, and 2–4 were fitted to a GLM with a Poisson distribution. We also tested whether there is an association between offspring development time (i.e., the time between larval arrival on the carcass and dispersal) and care duration using a GLM with a Poisson distribution.

For our analyses of offspring performance, we tested the effect of initial brood size, carcass size and male size on the average weight of dispersing larvae using a GLM with a Gaussian distribution, and on the larval survival rate till dispersal as well as from dispersal to eclosion using quasi binomial GLMs. Again, the interaction between carcass size and initial brood size was included in the models. Additionally, we calculated GLMs to investigate if male care duration, the absolute amount of direct care and the absolute amount of indirect care affected the average larval weight, the survival rate of larvae till dispersal and the larval survival rate till adulthood. Since the total number of observations per brood depended on the time of offspring dispersal and therefore varied between broods, we additionally calculated the relative amount of direct/indirect care by dividing their amounts by the sum of all observations. In separate GLMs we analyzed the effect of the relative amount of direct/indirect care on the average larval weight and the survival rate of larvae till dispersal and from dispersal to eclosion. The analyses involving the duration and amount of care were conducted in separate GLMs because of a collinearity between these predictor variables (as well as between them and male body size). However, to examine whether any effects of the duration and amount of care on offspring performance depended on initial brood or carcass size, we re‐ran all the models and included brood size, carcass size and the interaction between brood and carcass size as fixed effects.

All F‐, *χ* ^2^‐ and *p*‐values provided in the text and the tables were obtained using the “Anova” function of the R package ‘*car*’ (Fox & Weisberg, 2017). In addition, we calculated R^2^ for generalized linear models in the R package ‘*rsq*’ (Zhang, 2018). We furthermore performed post hoc tests using the “emmeans” or the “emtrends” (for comparisons of slopes) function in the ‘*emmeans*’‐package (Lenth, 2019), if carcass size or the interaction between carcass size and initial brood size showed significant effects in our models. *p*‐values were adjusted for multiple comparisons using the Tukey‐method.

## 3 RESULTS

### 3.1 The impact of initial brood, carcass, and body size on male care decisions

**FIGURE 1:**
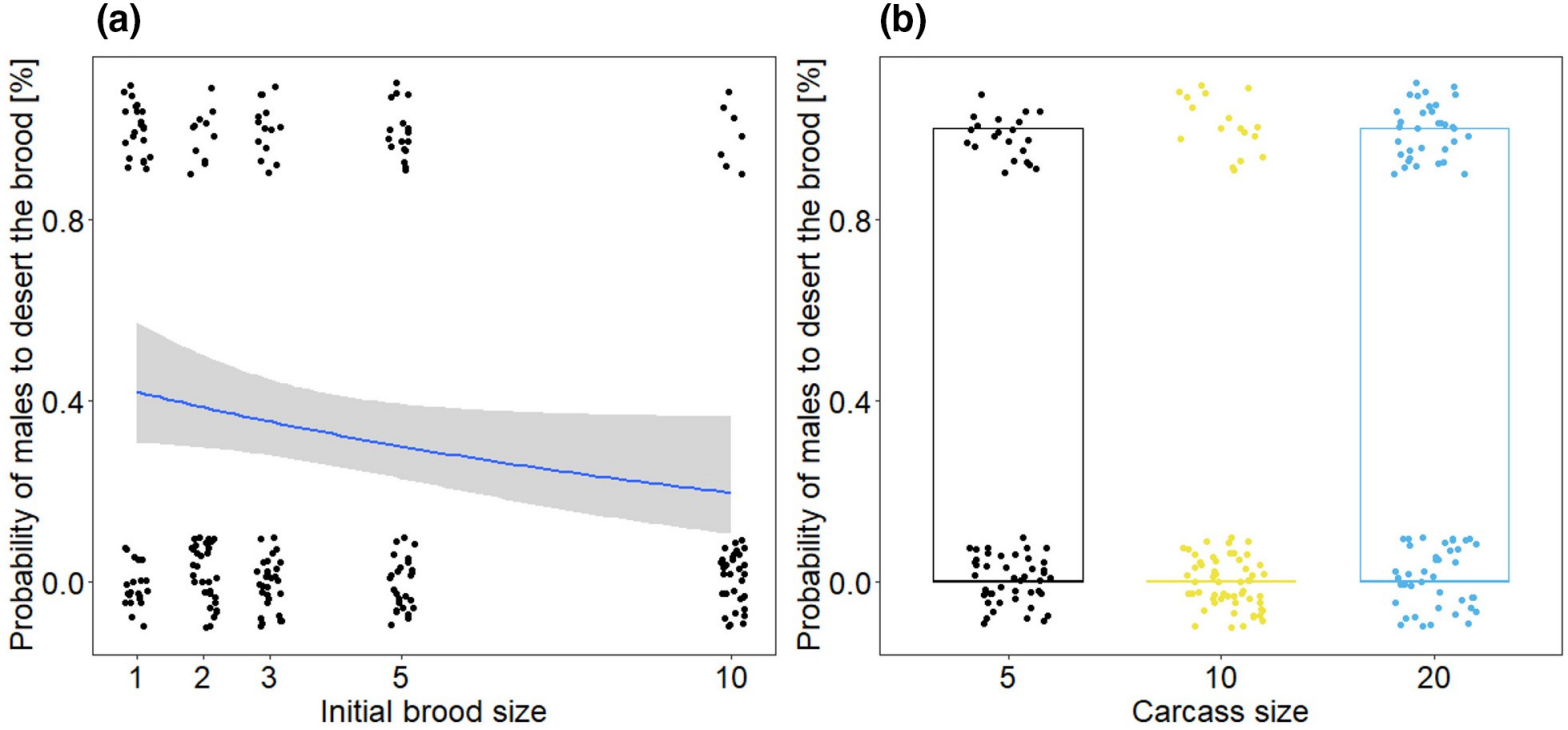
Relationship between the probability of *N. vespilloides* males to desert their given brood and (a) the initial brood size and (a) the carcass size. The dots represent the original data, the lines represent the calculated regression lines and their respective 95% CI.

From the 213 males, only 4 were never observed on the carcass after providing them with larvae. Most males (*N* = 141) remained with the brood until larval dispersal and 71 males engaged in parenting but deserted the brood earlier. The probability of offspring desertion decreased with increasing initial brood size (Table 1, Figure 1a). The highest percentage of offspring desertion was found with 1 larva as initial brood (48.8%), while the lowest desertion of larvae was observed when males obtained 10 larvae to care for (15%). Further, the frequency of offspring desertion was affected by carcass size (Table 1; Figure 1b). We found that 26.15% of males abandoned their offspring at 5 g, 18.92% of males deserted at 10 g and 37.84% left their offspring at 20 g carcasses (Table S1). Neither the male size nor an interaction between carcass and initial brood size showed an effect on male desertion (Table 1).

**TABLE 1:** Summary of the model on the effects of brood size, carcass size, male pronotum size and the interaction between carcass size and brood size on the probability of offspring desertion (*R* ^2^ = .09) and the care duration (*R* ^2^ = .02).

Predictors	Offspring desertion			Care duration		
	*χ* ^2^	df	*p*	*χ* ^2^	df	*p*
Initial brood size	4.04	1	**.04**	0.51	1	.47
Carcass size	9.73	2	**.008**	25.76	2	**<.001**
Male size	0.25	1	.62	28.15	1	**<.001**
Carcass size × brood size	3.75	2	.15	13.22	2	**.001**

*Note*: Significant values are in bold.

**FIGURE 2:**
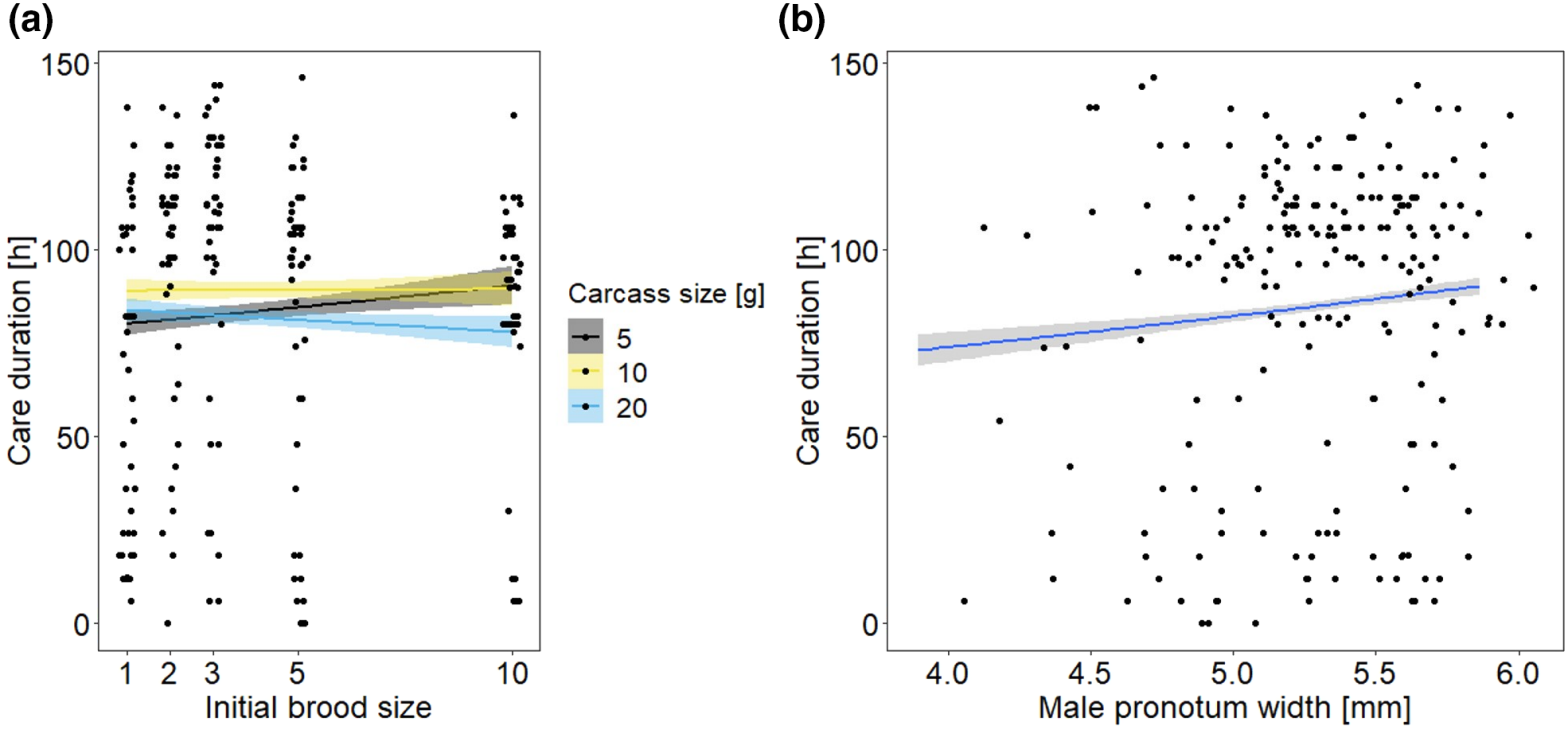
Relationship between the care duration of males and (a) the initial brood size on three different carcass sizes and (a) the size of males. The lines show the calculated regressions and their 95% CI, while the dots show the original data.

The duration of male care was not affected by the initial brood size, but carcass size showed an effect (Table 1). More importantly, the interaction between carcass and initial brood size was significant (Table 1). On 5 g carcasses, care duration slightly increased with increasing initial brood size, on 10 g carcasses it remained constant across brood sizes and on 20 g carcasses, the time males spent with the brood decreased with increasing brood size (Figure 2a). Slopes differed significantly between 5 and 10 g carcasses and between 5 and 20 g carcasses (Table S2). Male size affected care duration (Table 1), with larger males caring longer than smaller ones (Figure 2b). We also found an association between offspring development time and care duration, with males remaining longer on the carcass when offspring dispersed later (GLM, *χ* ^2^_1,212_ = 1242.7, *p* < .001).

**FIGURE 3:**
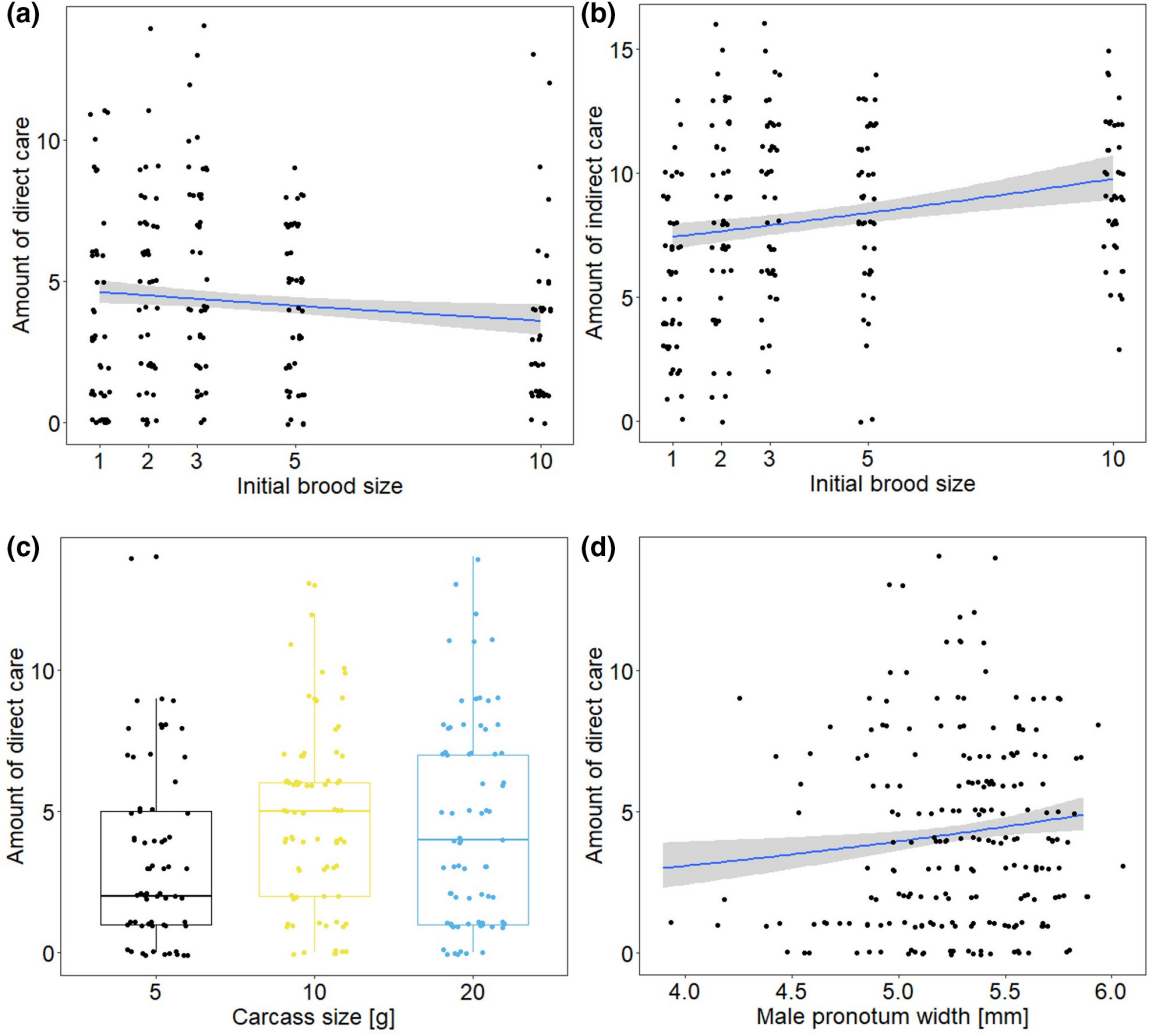
Relationship between the amount of (a) direct care and (b) indirect care with the initial brood size. (c) shows the relationship between the amount of direct care and the carcass size, and (d) the male size. The amount of direct care is the number of observations in which the male was found at or in the feeding cavity. The amount of indirect care is the number of observations in which the male was found at the carcass but not at or in the feeding cavity. The lines show the calculated regressions and their 95% CI, while the dots show the original data.

The amount of direct as well as indirect care was affected by the initial brood size. Surprisingly, males provided less direct care and more indirect care with increasing brood size (Table 2, Figure 3a,b). The size of the carcass had only an effect on the amount of direct care (Table 2), with males showing more direct care with intermediate and large carcass size than on small carcasses (Table S3, Figure 3c). There was no interaction effect between initial brood size and carcass size on both types of care (Table 2). Larger males provided direct care more often than smaller males (Table 2; Figure 3d). Body size had no effect on the amount of indirect care (Table 2).

**TABLE 2:** Summary of the model on the effects of initial brood size, carcass size, male pronotum size and the interaction between carcass size and brood size on the amount of direct care (*R* ^2^ = .07) and indirect care (*R* ^2^ = .06) of males.

Predictors	Direct care			Indirect care		
	*χ* ^2^	df	*p*	*χ* ^2^	df	*p*
Initial brood size	6.19	1	**.01**	17.83	1	**<.001**
Carcass size	20.94	2	**<.001**	0.99	2	.61
Male size	10.03	1	**.001**	0.01	1	.92
Carcass size × brood size	5.25	2	.07	2.65	2	.26

*Note*: Significant values are in bold.

### 3.2 Offspring performance

**FIGURE 4:**
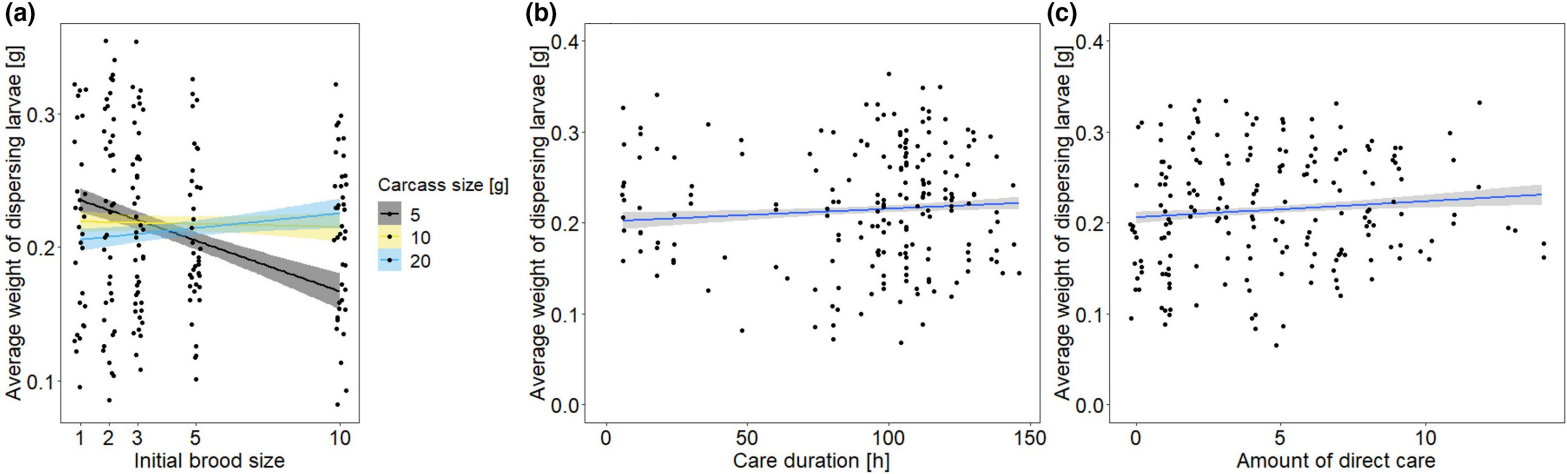
Relationship between the average weight of dispersing larvae and (a) the initial brood size on three different carcass sizes, (b) the care duration of males, and (c) the amount of direct care. The amount of direct care is the number of observations in which the male was found at or in the feeding cavity. Shown are the calculated regression lines and their respective 95% CI. The dots represent the original data.

We found that the average weight of the dispersing larvae decreased with increasing brood size (Table 3). This effect, however, depended on carcass size (Table 3). On 5 g carcasses, the average weight of dispersing larvae decreased with increasing initial brood size, whereas the average larval weight slightly increased on 10 and 20 g carcasses (Figure 4a). Slopes differed significantly between 5 and 10 g carcasses and between 5 and 20 g carcasses (Table S4). Male size showed no effect on the mean larval weight at dispersal (Table 3). We found that with increasing male care duration (GLM, *F*_1,191_ = 7.49, *p* = .007, Figure 4b) and with a higher absolute amount of direct care (GLM, *F*_1,191_ = 9.94, *p* = .002, Figure 4c), the dispersing larvae showed a higher average weight. Looking at the relative amount of direct care, we also found a positive effect on average larval weight (GLM, *F*_1,191_ = 5.85, *p* = .02). Neither the absolute (GLM, *F*_1,191_ = 2.1, *p* = .15) nor the relative amount of indirect care (GLM, *F*_1,191_ = .53, *p* = .47) showed an effect on the average larval weight.

**TABLE 3:** Summary of the model on the effects of initial brood size, carcass size, male pronotum size and the interaction between carcass size and brood size on the average larval weight at dispersal (*R* ^2^ = .27) and the larval survival until dispersal (*R* ^2^ = .06).

Predictors	Average larval weight			Survival until dispersal		
	*F*	df	*p*	*F*	df	*p*
Initial brood size	8.01	1	**.005**	6.21	1	**.01**
Carcass size	1.32	2	.27	0.46	2	.63
Male size	0.06	1	.8	7.83	1	**.006**
Carcass size × brood size	28.17	2	**<.001**	0.17	2	.84

*Note*: Significant values are in bold.

**FIGURE 5:**
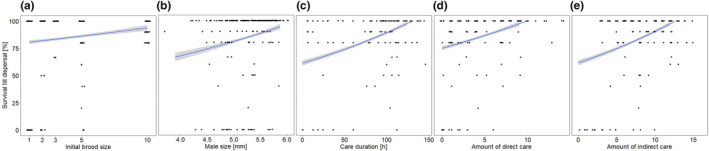
Relationship between the larval survival rate until dispersal and (a) the initial brood size, (b) the size of males, (c) the duration of care, (d) the amount of direct care by males, and (e) the amount of indirect care from males. The amount of direct care is the number of observations in which the male was found at or in the feeding cavity. The amount of indirect care is the number of observations in which the male was found at the carcass but not at or in the feeding cavity. Shown are the calculated regression lines and their respective 95% CI. The dots represent the original data.

Larval survival rate until dispersal (Table 3, Figure 5a) and from dispersal to eclosion (Table S5, Figure S1a) was higher with increasing brood size. Carcass size had no effect on the survival rate till dispersal (Table 3) and from dispersal to eclosion (Table S5). We found no effect of the interaction between the initial brood and carcass size on survival rate until dispersal (Table 3) and from dispersal to eclosion (Table S5). Larval survival rate until dispersal was higher if the caring male was larger (Table 3, Figure 5b). Male size, however, did not affect larval survival rate from dispersal to eclosion (Table S5).

The duration of male care had a positive effect on the survival rate of larvae until dispersal (GLM, *F*_1,212_ = 39.63, *p* < .001, Figure 5c) and from dispersal to eclosion (GLM, *F*_1,156_ = 24.44, *p* < .001, Figure S1b). The absolute amount of direct as well as indirect care had a positive effect on larval survival until dispersal (direct: GLM, *F*_1,212_ = 22.17, *p* < .001, Figure 5d; indirect: GLM, *F*_1,212_ = 38.78, *p* < .001, Figure 5e) and from dispersal to eclosion (direct: GLM, *F*_1,156_ = 7.87, *p* = .006, Figure S1c; indirect: GLM, *F*_1,156_ = 39.21, *p* < .001, Figure S1d). Further, the relative amount of direct care had a positive effect on the larval survival until dispersal (GLM, *F*_1,212_ = 7.55, *p* = .006) but no effect on the survival rate from dispersal to eclosion (GLM, *F*_1,156_ = 0.85, *p* = .36). In contrast, the relative amount of indirect care had neither an effect on the survival rate until dispersal (GLM, *F*_1,212_ = 3.33, *p* = .07) nor from dispersal to eclosion (GLM, *F*_1,156_ = 2.63, *p* = .11).

When we included brood and carcass size in all the models that tested for effects of duration or amount of care on offspring performance, all the significant effects remained with one exception: the effect of the relative amount of direct care on average larval weight was not significant anymore.

## 4 DISCUSSION

In this study, we investigated factors influencing the care strategies of uniparental males and their downstream consequences on offspring performance. By analyzing more than 200 burying beetle families, we found that initial brood size, carcass size and male body size affected the amount and type of care males provided. Furthermore, we found that males that cared longer and showed a higher amount of direct care raised more and heavier larvae. Thus, our study shows that uniparental males are plastic in their care strategies, adjusting the amount and type of care behavior to the value of the brood and the resource as well as their own quality, and these adjustments appear to have consequences for offspring survival and fitness.

Our first main finding was that a surprisingly high number of males stayed and cared for a given brood until larval dispersal and this despite the small initial brood sizes used in the experiment. Even when only one larva arrived at the carcass, about half of the males decided to provide care and did not desert the brood. The reason for this is likely the low abundance of suitable breeding resources in nature. Small vertebrate cadavers are nutrient rich but ephemeral and unpredictably distributed resources. The probability to find a new cadaver and monopolize it is low, which makes staying on an already found breeding resource and raising a given brood likely a beneficial strategy. Moreover, previous studies revealed that males also benefit personally from remaining with the brood because they themselves can feed from the carrion resource. This, in turn increases their attractiveness to females as they are able to produce a higher quantity of their sex pheromone after having reared a brood (Chemnitz et al., 2017; Keppner & Steiger, 2021).

Further we found that brood size affected male care strategies. Males deserted a brood less frequently and increased their amount of indirect care with increasing brood size. This suggests that uniparental *N. vespilloides* males – similar to females (Sahm et al., 2022) ‐ can estimate the value of the brood and make decisions based on it. Our result is in line with a previous study which also showed that uniparental males abandoned smaller broods more frequently than larger broods (Ward et al., 2009). In general, our result adds to growing evidence that males are sensitive to social cues and invest according to the size and therefore the value of a brood. A brood‐size dependent male care strategy has, for example, also been found in snail kites (Beissinger, 1990), in bluegill sunfish (Coleman et al., 1985; Coleman & Fischer, 1991) or in sand gobies (Forsgren et al., 1996). Surprisingly, although we found that males increased their amount of indirect care with increasing brood size, brood size had a negative effect on the amount of direct care. We would have expected that uniparental males ‐ similar to uniparental females or biparental males – spent more time provisioning larvae when brood size increased (Rauter & Moore, 2004; Smiseth et al., 2007; Smiseth & Moore, 2004a; Wang, Ma, et al., 2021; Wang, Zhou, et al., 2021). Also, in many birds increasing brood size usually results in an increased provisioning rate by parents (Ardia, 2007; García‐Navas & Sanz, 2010; Neuenschwander, 2003). One explanation for our result might be that we did not distinguish if males provisioned themselves or their offspring when they visited the feeding cavity (scored as direct care). To save more food for their offspring, it is possible that uniparental males reduce the amount of carrion consumed by themselves with increasing brood size by visiting the feeding cavity less often.

Our next significant finding was that male care strategies were affected by carcass size. Males deserted more frequently from large carcasses than from intermediate sized carcasses. They also provided more direct care on intermediate carcasses than on small carcasses. A likely explanation for these findings is that tending broods on intermediate sized carcasses results in the best cost–benefit ratio of care. Small carcasses have less food available and can lead to a low‐quality brood. Large carcasses, on the other hand, might be very costly to maintain and defend, making it unprofitable to raise broods of small sizes. That larger carcasses are more costly to prepare was also suggested in previous studies (de Gasperin & Kilner, 2015; Ratz et al., 2021; Xu & Suzuki, 2001). For example, de Gasperin and Kilner (2015) found that the preparation of larger carcasses resulted in a reduced lifespan of male beetles. In general, our results highlight that males are able to evaluate resource size and are consistent with the results of previous studies that examined male care behavior under biparental care. Bartlett (1988), Kishida and Suzuki (2010) and Ratz et al. (2021), for example, found that males are sensitive to carcass size and leave the brood earlier as carcass mass decreases. Males of other species are also known to monitor resource availability (e.g., Barbasch et al., 2020) or other non‐social environmental factors (e.g., Green & McCormick, 2004) and adjust care behavior accordingly. For example, male glass frogs increase both the frequency and the amount of time spent incubating eggs when humidity is declining (Delia et al., 2013).

Another key finding of our study was that male care strategies depended on a male's body size, albeit the effects were relatively small. Larger males cared longer and provided more direct care than smaller males. This observation is consistent with the idea that larger males suffer lower costs from maintaining the carcass and caring for the brood or have a greater benefit from doing so. A previous study on females found that larger females were able to raise heavier larvae, likely because they have a greater capacity to feed the offspring (Steiger, 2013). Likewise, larger males might be able to ingest, process and regurgitate a higher amount of food, leading to a higher larval mass and making it more profitable to defend the brood for a longer time than smaller males. Although we could not find any effect of male body size on offspring mass, we found a positive effect on larval survival until dispersal, a result that is in line with our hypothesis. However, based on our study it is impossible to disentangle cause and consequences. Males might stay longer because they have a higher reproductive output, but it is also possible that the higher reproductive output is the consequence of their prolonged stay. Furthermore, there are also alternative explanations for our results. Larger males might have a higher chance to defend the brood and resource from intruders and therefore larger males might tend to stay longer with their brood. This seems likely, as previous studies in burying beetles found that larger males are indeed predominantly the winners in competitions over resources (Bartlett & Ashworth, 1988; Luzar et al., 2017; Otronen, 1988). We also need to consider the possibility that larger males remained longer and showed a higher presence in the feeding cavity (scored as direct care) because larger males need to consume more carrion food to replenish their energy reserves. In fact, Pilakouta et al. (2016) showed that larger parental beetles spent more time feeding from the carcass and gained more weight during the breeding event than smaller ones. Whether the prolonged stay of larger males is due to selfish reasons or for the benefit of the larvae needs to be evaluated in future studies. Interestingly, a study of Smith et al. (2014) found an opposite effect of male body size on residency time, with smaller males remaining longer with the brood than larger ones. The seemingly contradictory result might simply be explained by species differences in the effect of body size on care duration or residency time: the study of Smith et al. (2014) focused on *N. orbicollis*, a species of larger average body size than *N. vespilloides*. However, it is also possible that the effect varies between populations and depends on the local intensity of inter‐ and intraspecific competition for carrion resources and the availability of mating partners. Recent studies found that even populations in close proximity can differ in their mean body size and breeding strategy, likely caused by differences in population densities and community structure (Sun et al., 2020). In general, if male size determines the ability to secure further mating or breeding opportunities, the importance of an individual's size for paternal care decisions might vary according to the abundance of potential mates or breeding resources (see also Robertson & Roitberg, 1998).

Concerning offspring performance under uniparental male care, we found that the interaction between initial brood and carcass size showed a significant effect on offspring mass. On small carcasses brood size had a negative impact on larval mass, whereas on larger carcasses brood size had a positive effect. This result is in line with a study of Schrader et al. (2015), which found that brood size had a beneficial effect on larval mass at lower larval densities (i.e. number of larvae per gram carcass) but a detrimental effect on larval mass at higher larval densities. Schrader et al. (2015) argued that this likely reflects a density dependent shift from sibling cooperation to competition. If the number of larvae per gram carcass is very high, the larvae inevitably compete for food, because there is a limited amount of food available for each larva. If larval density is low, they benefit from having siblings because collectively they are more efficient in utilizing the resource (Prang et al., 2022). However, currently there is only mixed evidence for sibling cooperation in *N. vespilloides*. A study of Magneville et al. (2018), for example, did not find any positive effect of brood size on offspring performance and Prang et al. (2022) only found signs of sibling cooperation when larvae developed on a parentally unprepared carcass. Interestingly, the study of Schrader et al. (2015) found a positive effect of brood size on offspring mass only in the absence of parents, but not under biparental care. Here, we revealed a positive effect of brood size on offspring mass under uniparental male care. Moreover, not only offspring mass but also offspring survival increased with brood size. However, based on our data we currently cannot say whether the positive effect of brood size on offspring performance is caused by sibling cooperation. It is also possible that males invest more in care when confronted with larger broods. In fact, although our own study did not find any positive effect of brood size on care duration or the amount of direct care, we found that males were less likely to desert larger broods and showed more indirect care. It is also possible that males actively cannibalized small unrentable broods, as we had some broods with no surviving larvae. Irrespective of the underlying mechanisms – which must be studied in more detail ‐ our result shows that under male care, offspring benefit from having siblings when the carcass is large enough. This result has important wider implications for our understanding of the evolution of family life. When it comes to sibling interactions past studies have predominantly focused on sibling competition (Kramer & Meunier, 2018). However, siblings can profit from each other either because they actively cooperate, for example, by sharing food (Falk et al., 2014) or cleaning each other (Roulin et al., 2016) or because larger broods are able to extract a higher per‐capita investment from their parents than smaller ones.

Another key finding of our study was that offspring raised by larger males showed a higher survival rate until dispersal. We suggest two non‐exclusive hypotheses to explain this result. First, larger males might have a higher efficiency to allocate food to their offspring, as they might be able to predigest a higher amount of food in a specific time. This idea has also been proposed by Steiger (2013), who found that larger burying beetle females raised heavier offspring. In our current study, we found that larger males cared longer and showed a higher amount of direct care. Hence, a second possibility is that the higher survival rate is simply the consequence of the higher amount of time invested in care. That larger males are better fathers has also been found in other species. For example in biparental dung and tenebrionid beetles, females assisted by larger males produced heavier offspring (Heg & Rasa, 2004; Hunt & Simmons, 2000) and in a sand goby, larger males lost less eggs during egg guarding than smaller ones (Hunt & Simmons, 1998; Lindström & Hellström, 1993).

Finally, our study showed that an increased amount of direct and indirect care and an increased care duration of uniparental males correlated positively with larval mass and survival. This result indicates that the males do not only remain with the carrion resource due to personal benefits and confirms that the variables we measured indeed reflects parental care, i.e., parental traits that enhance offspring fitness. That offspring performance is positively linked to the amount of male care has also been shown in other taxa. For example, in a Neotropical glass frog, early male removal resulted in a higher embryo mortality due to dehydration than later removals. Also in a range of biparental birds (see Bart & Tornes, 1989 and references therein) and in the biparental California mouse, *Peromyscus californicus* (Gubernick & Teferi, 2000), male removal reduced offspring survival.

It has been widely accepted that parenting strategies can be complex and flexibly adjusted to the social and non‐social environment as well as to the caregiver's own quality or condition (Royle et al., 2014; Royle & Hopwood, 2017). However, how responsive parents are, depends on how strong selection has acted on behavioral plasticity in the past. Furthermore, a high responsiveness towards the social environment might limit the plasticity of care behavior towards the non‐social environment and vice versa (Royle et al., 2014). Here we found that even though male uniparental care is thought to be rare in nature, burying beetle males are very responsive and adjust their care strategies to both their social (offspring) as well as their non‐social (carrion resource) environment. They even base their decisions on their own body size. Earlier studies also found that males are plastic in their response, providing more care in the absence than in the presence of females (Bartlett, 1988; Moss & Moore, 2021; Royle et al., 2014; Smiseth et al., 2005). These results, together with the previous finding that male uniparental care is as effective as female uniparental care (Parker et al., 2015), might indicate that male uniparental care occurs more frequently than previously thought. However, there are also signs that males are not as responsive as females to offspring need (Moss & Moore, 2021; Royle & Hopwood, 2017; Suzuki & Nagano, 2009), suggesting that selection on plasticity acts much stronger on the primary caregiver, likely because higher offspring contact can promote the evolution of fine‐tuned parent‐offspring communication. In general, who cares and to which degree is very flexible in burying beetles, but which factors promote or impede flexible parenting strategies in caring species is largely unknown and requires further research. We especially need more studies quantifying the level of responsiveness exhibited by both male and female parents. To this end, the removal of the female parent or the primary caregiver – as done in our study – could be a valuable tool to reveal how responsive the secondary caregiver is towards environmental or intrinsic cues. This is because the presence of the primary caregiver likely masks or limits the displayed responsiveness of the second parent.

## AUTHOR CONTRIBUTIONS

**Jacqueline Sahm:** Conceptualization (equal); investigation (lead); visualization (lead); writing – original draft (lead). **Taina Conrad:** Writing – original draft (supporting). **Larissa Scheu:** Investigation (supporting). **Sandra Steiger:** Conceptualization (equal); funding acquisition (lead); resources (lead); supervision (lead); writing – original draft (supporting).

## CONFLICT OF INTEREST STATEMENT

The authors declare no competing interests.

## Supporting information


Appendix S1.

## Data Availability

Data will be submitted to DRYAD. 10.5061/dryad.bnzs7h4gk.
