# Supplementary material for: Brood size, food availability, and body size affects male care decisions and offspring performance
Source: Ecol Evol. 2023 Jun 8;13(6):e10183. doi: 10.1002/ece3.10183 (PMC10249043; doi:10.1002/ece3.10183)
Supplement: Supplementary file 1 — Appendix S1. [file ECE3-13-e10183-s001.docx]

**Supporting Information**

**Brood size, food availability, and body size affects male care decisions and offspring performance**

Jacqueline Sahm^1,*^, Taina Conrad^1^, Larissa Scheu^1^, Sandra Steiger^1^

**Table S1:** Post hoc pairwise comparisons for the effect of carcass size (5 g, 10 g, 20 g) on the probability of offspring desertion.

| response | predictor | estimate (±SE) | z-ratio | p-value |
| --- | --- | --- | --- | --- |
| offspring desertion | carcass size |  |  |  |
|  | 10 vs 20 | -1.18 (0.37) | -3.17 | **0.004** |
|  | 10 vs 5 | -0.56 (0.39) | -1.42 | 0.33 |
|  | 20 vs 5 | 0.63 (0.36) | 1.73 | 0.2 |

| response | predictor | estimate (±SE) | z-ratio | p-value |
| --- | --- | --- | --- | --- |
| care duration | carcass size x brood size |  |  |  |
|  | 10 vs 20 | 0.01 (0.006) | 1.26 | 0.42 |
|  | 10 vs 5 | -0.01 (0.06) | -2.44 | **0.04** |
|  | 20 vs 5 | -0.02 (0.06) | -3.59 | **0.001** |

**Table S2:** Post hoc pairwise comparisons for the interaction effect of carcass size (5 g, 10 g, 20 g) and initial brood size on care duration (i.e., level-wise comparisons of slopes).

**Table S3:** Post hoc pairwise comparisons for the effect of carcass size (5 g, 10 g, 20 g) on the amount of direct care.

| response | predictor | estimate (±SE) | z-ratio | p-value |
| --- | --- | --- | --- | --- |
| direct care | carcass size |  |  |  |
|  | 10 vs 20 | 0.02 (0.08) | 0.25 | 0.97 |
|  | 10 vs 5 | 0.36 (0.09) | 4.19 | **<0.001** |
|  | 20 vs 5 | 0.34 (0.09) | 3.93 | **<0.001** |

| response | predictor | estimate (±SE) | z-ratio | p-value |
| --- | --- | --- | --- | --- |
| average larval weight | carcass size x brood size |  |  |  |
|  | 10 vs 20 | -0.002 (0.001) | -2.07 | 0.1 |
|  | 10 vs 5 | 0.007 (0.001) | 5.32 | **<0.001** |
|  | 20 vs 5 | 0.01 (0.001) | 7.36 | **<0.001** |

**Table S4:** Post hoc pairwise comparisons for the interaction effect of carcass size (5 g, 10 g, 20 g) and initial brood size on average larval weight (i.e., level-wise comparisons of slopes).

**Table S5:** Summary of the GLM predictors affecting the survival of larvae from dispersal to eclosion (R² = 0.19). Significant values are in bold.

| Predictors | Survival to eclosion | | |
| --- | --- | --- | --- |
|  | F | df | p |
| Initial brood size | 10.02 | 1 | **0.002** |
| Carcass size | 2.86 | 2 | 0.06 |
| Male size | 1.71 | 1 | 0.19 |
| Carcass size x brood size | 0.51 | 2 | 0.6 |


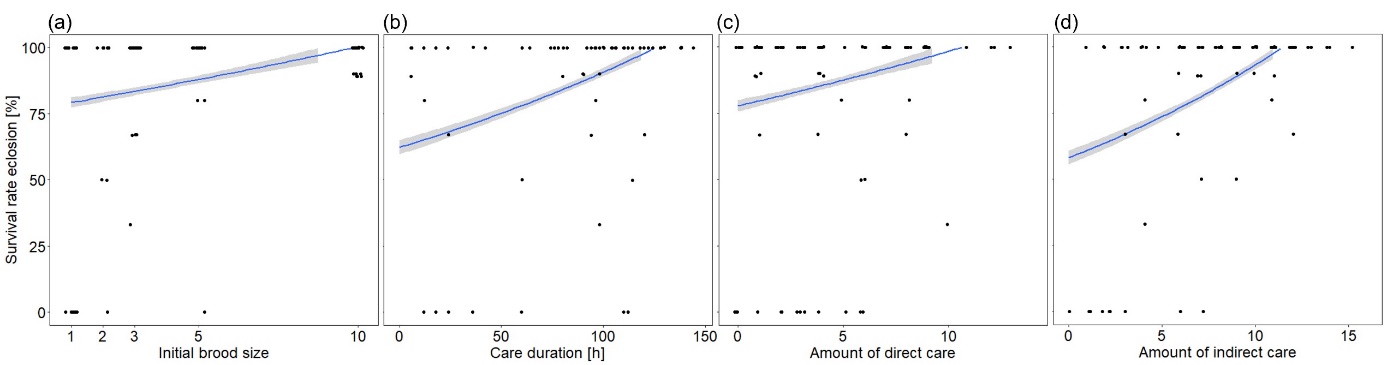


**Figure S1:** Relationship between the larval survival rate from dispersal to eclosion and (a) the initial brood size, (b) the care duration, (c) the amount of direct care and (d) the amount of indirect care. The amount of direct care is the number of observations in which the male was found at or in the feeding cavity. The amount of indirect care is the number of observations in which the male was found at the carcass but not at or in the feeding cavity. The dots represent the original data and we calculated regression lines and their respective 95% CI.
